# Validation of the Dutch-Flemish translated ABCD questionnaire to measure cardiovascular diseases knowledge and risk perception among adults

**DOI:** 10.1038/s41598-021-88456-5

**Published:** 2021-04-26

**Authors:** Hamid Yimam Hassen, Naomi Aerts, Stefaan Demarest, Md Dilshad Manzar, Steven Abrams, Hilde Bastiaens

**Affiliations:** 1 Department of Primary and Interdisciplinary Care, Faculty of Medicine and Health Sciences, University of Antwerp, Doornstraat 331, Wilrijk, 2610 Belgium; 2 Global Health Institute, Faculty of Medicine and Health Sciences, University of Antwerp, Doornstraat 331, Wilrijk, 2610 Belgium; 3 Interuniversity Institute for Biostatistics and Statistical Bioinformatics, Data Science Institute, Hasselt University, Diepenbeek, Belgium; 4 Department of Public Health and Surveillance, Scientific Institute of Public Health, Juliette Wytsmanstraat 14, 1050 Brussels, Belgium; 5 Department of Nursing, College of Applied Medical Sciences, Majmaah University, Al Majmaah, Saudi Arabia

**Keywords:** Cardiology, Health care, Medical research

## Abstract

Valid and reliable measurement of an individual’s knowledge and risk perception is pivotal to monitor and evaluate the effectiveness of interventions aimed at preventing cardiovascular diseases (CVDs). The recently developed Attitudes and Beliefs about Cardiovascular Disease (ABCD) knowledge and risk questionnaire is shown to be valid in England. In this study, we evaluated the psychometric properties of the modified and Dutch (Flemish)-translated ABCD questionnaire using both the classical test and item response theory (IRT) analysis. We conducted a community-based survey among 525 adults in Antwerp city, Belgium. We assessed the item- and scale-level psychometric properties and validity indices of the questionnaire. Parameters of IRT, item scalability, monotonicity, item difficulty and discrimination, and item fit statistics were evaluated. Furthermore, exploratory and confirmatory factorial validity, and internal consistency measures were explored. Descriptive statistics showed that both the knowledge and risk scale items have sufficient variation to differentiate individuals’ level of knowledge and risk perception. The overall homogeneity of the knowledge and risk subscales was within the acceptable range (> 0.3). The exploratory and confirmatory factor analyses of the risk scale supported a three-factor solution corresponding to risk perception (F1), perceived benefits and intention to change physical activity (F2), and perceived benefit and intention to change healthy dietary habit (F3). The two parametric logistic (2—PL) and rating scale models showed that the item infit and outfit values for knowledge and risk subscales were within the acceptable range (0.6 to 1.4) for most of the items. In conclusion, this study investigated the Dutch (Flemish) version of the ABCD questionnaire has good psychometric properties to assess CVD related knowledge and risk perception in the adult population. Based on the factor loadings and other psychometric properties, we suggested a shorter version, which has comparable psychometric properties.

## Introduction

Cardiovascular diseases (CVDs) are the leading cause of adult morbidity and mortality worldwide, accounting for an estimated 18.6 million deaths and 393 million disability adjusted life years (DALYs) in 2019^1,2^. Similarly, 4 million deaths each year and 25% of all-cause DALYs in Europe are due to CVDs, so being the major cause of disease burden than any other condition^3,4^. The proportion of total deaths due to CVD greatly varies among countries in Europe, with over a twofold difference between Bulgaria (62%) and France (26%)^5^. In Belgium, CVDs account for 28% of all deaths and 14.2% of total DALYs^4,6^.

The relationship between lifestyle and CVD is well established. Physical inactivity, unhealthy dietary habit, tobacco use, excessive alcohol consumption, and stress have all been identified as independent risk factors of CVD^7–9^. The adoption of a healthy lifestyle has also been shown to reduce CVD risk^10^. In this regard, active involvement of the target population is crucial for CVD preventive interventions aiming at improving such healthy lifestyles. Likewise, knowledge of behavioral risks is essential for behavior change and individuals who perceive themselves to have a higher risk of CVD are more likely to adopt a healthy lifestyle^11,12^. Moreover, knowledge of the risk factors in comparison with one’s own lifestyle may form the risk perception^13^, which is a central psychological construct that could affect adoption of healthy lifestyle and maintenance^14,15^. As a result, improving knowledge and risk perception is an integral part of behavioral interventions aimed at reducing incidence of CVD^15–17^.

Poor knowledge and the gap between actual risk and the risk perception of the general population impedes the attainment of better health outcomes. Therefore, measurement of a person’s CVD knowledge and risk perception is essential to develop a healthy lifestyle intervention and to evaluate undergoing preventive activities. However, valid and reliable tools to measure CVD knowledge and risk perception among the adult population are scant. Few studies assessed risk perception using a single question^18,19^, which is not sufficient to assess multiple dimensions thereof^20^. The Heart Disease Fact Questionnaire (HDFQ) is put forward as a comprehensive measure of CVD risk knowledge, however, the focus is on measuring knowledge among patients with diabetes and it does not measure the risk perception^21^. In response, Woringer et al. developed a questionnaire to measure CVD knowledge and risk perception in England^22^. The study used an extensive scale development procedure and found a significant correlation between perceived risk measured by the questionnaire and predicted risk of CVD. Moreover, the tool has shown to have acceptable psychometric validity to be used in practice. They named the questionnaire Attitudes and Beliefs about Cardiovascular Disease Risk Questionnaire, or in short the ABCD questionnaire. The questionnaire consists of 26 items grouped into four scales: Knowledge of CVD Risk and Prevention (eight items), Perceived Risk of Heart Attack/Stroke (eight items), Perceived Benefits and Intention to Change Behavior (seven items) and Healthy Eating Intentions (three items).

The multi-country CVD prevention study named ‘SPICES’—Scaling-up Packages of Interventions for Cardiovascular diseases in Europe and Sub-Saharan Africa identified the ABCD questionnaire as a potential instrument to measure CVD knowledge and risk perception prior to and after the planned intervention. However, the questionnaire was not translated and validated in the Belgium context. Therefore, this study aimed to psychometrically investigate a modified and Dutch (Flemish) translated ABCD questionnaire, in terms of validity and reliability, using both the classical test (CTT) and item response theory (IRT) approach.

## Methods

### Study setting

This study is part of the ongoing SPICES project, and we particularly used data collected during the baseline population survey in the city of Antwerp, Belgium. The city of Antwerp is subdivided into 9 districts with over half a million inhabitants. The SPICES project in Belgium targets a vulnerable population based on the principle of ‘proportionate universalism’, in which assessments and interventions are universal, not targeted, but with a scale proportionate to the level of vulnerability^23^. Hence, instead of stratifying people on an individual level vulnerability, we rather focused on target ‘vulnerable districts’ based on the socioeconomic deprivation index, access to primary healthcare, density of households with social support, and density of elderly inhabitants. Using the aforementioned criteria, two districts, Deurne Noord and Borgerhout Intramuros, were selected.

### Study sample and procedure

Data were collected using postal and online platforms on randomly selected adults in the two selected districts of Antwerp from February to July 2020. We obtained a stratified random sample of inhabitants from the Antwerp city administration based on the probability proportional to size (PPS) sampling technique. The stratification was based on age group (5-year interval), sex and statistical sector (the lowest administrative unit). The inclusion criteria were: being a registered resident of Antwerp and aged 18 years or older. To remediate a high non-response rate, we used a matched substitution technique for non-respondents, which is shown to have acceptable external validity^24^. Substitutes were matched based on age, sex and geographical location, in substitution phases. In order to increase the response rate, we sent a reminder for participants who did not respond within ten days as recommended by several studies^25,26^. Out of 1,512 invited, 543 participants responded to the survey. During data processing, four questionnaires indicated that the participant could not fill it due to cognitive impairment (noted on the questionnaire), 14 participants had missing responses in all the required items, hence, leading to a total of 525 participants included in this validation analysis.

### Measures

#### Adaptation of the original ABCD questionnaire

We made minor modifications to the original ABCD questionnaire, next to the translation to Dutch (Flemish). The knowledge scale in the original tool has 8 items. We added one item about smoking “*People who smoke have an increased risk of cardiovascular diseases*”. In addition, question 7 in the original questionnaire was phrased as “*HDL refers to ‘good’ cholesterol, and LDL refers to ‘bad’ cholesterol*”. Hence, we modified this question into “*There is 'good' cholesterol and there is 'bad' cholesterol*” since the relevance of the English acronyms LDL and HDL in the questionnaire are considered to be minimal. Similarly in the risk scale, we added two items in order to have more insight on healthy diet intentions and phrased these items as “*I am considering eating at least 5 servings of fruit and vegetables a day* and “*I intend, or want, to eat at least 5 servings of fruit and vegetables a day*”.

#### The Dutch (Flemish) translated ABCD questionnaire

First, independent translation of the modified ABCD questionnaire was performed by language professionals who had experience in translation of medical questionnaires. Next, the Dutch (Flemish) version was back translated to English by experts in Linguapolis, University of Antwerp language institute. Then, the investigators discussed both versions of the questionnaire to check for consistency. Afterwards, a lay-man translation was performed to enhance understanding of the questionnaire. The knowledge scale consists of 9 statements about CVD risks, with response options being ‘true’, ‘false’ and ‘I don’t know’. For each item, the correct answer was scored as 1 and the incorrect or ‘I don’t know’ answers were both scored as 0. The Flemish version of the ABCD risk questionnaire consists of 20-items and response options were presented on a 4-point scale ranging from 1 ‘strongly disagree’ to 4 ‘strongly agree’, while items 6, 13 and 20 were reverse coded. Both the English and the Dutch (Flemish) translated questionnaire are available in the supplementary material.

### Statistical analyses

Upon checking for completeness, data were entered in the Research Electronic Data Capture (REDCap) database system. Then, we downloaded it as a CSV file and exported to the free R software package version 4.0.2 for further processing and analysis.

We followed a scale validation protocol for applied health research as suggested by Dima AL^27^. Descriptive statistics and Spearman rank correlations (rs) between items were examined. We evaluated the monotonicity and scalability of items using a Mokken Scaling Analysis (MSA)^28^. Coefficients of scalability (H), monotonicity and invariant item ordering were examined and H >  = 0.30 were considered to be scalable. As a rule of thumb, Mokken^28^ proposed a cutoff for H as follows: a scale is considered weak if 0.3 ≤ H < 0.4, moderate if 0.4 ≤ H < 0.5, and strong if H > 0.5.

To study the dimensionality of items of the risk scale in more detail, we performed an exploratory (EFA) and confirmatory factor analysis (CFA). We evaluated the sample and data adequacy for factor analysis using Kaiser–Meyer–Olkin (KMO) and Bartlett’s test of sphericity. EFA was performed using the maximum likelihood extraction and Oblimin rotation method on random half-sample (n = 262). We determined the adequate number of factors using a scree plot and parallel analysis. Then, to test the consistency of factors, we performed a CFA on the remaining half-sample (n = 263). We evaluated the model fit of the CFA using; the X^2^ test, the Tucker-Lewis and Comparative Fit Indexes and the root mean square error of approximation (RMSEA)^29^. Detailed procedures on EFA and CFA are available in the supplementary material.

We further examined the item properties of each scale using the parametric IRT analysis, which accounts for item difficulty and/or discrimination^30^. For the dichotomous knowledge scale, we fitted a Rasch model^31^ and a two-parameter logistic (2-PL) model, using conditional maximum likelihood (CML) estimation. We also provided a graphical illustration of the estimated item parameters using Item Characteristic Curves (ICCs). On the other hand, for the polytomous risk scale we used the Rating Scale Model, an extension of the Rasch model^32^. Diagnostics including item fit, global model fit, and joint ICCs were computed and results thereof are available in the supplementary material.

We computed the reliability analysis measures including Cronbach's alpha, Guttman’s lambda-6, beta, and omega. Furthermore, we compared psychometric properties of the short version we proposed with the original ABCD questionnaire.

### Ethics and participant consent

Along with the whole project, the protocol of this population survey was approved by the ethical committee of the University of Antwerp hospital (Approval No: B300201940009). Upon detailed description of study aim and procedures, informed consent was obtained from all study participants. The study was in accordance with the principles of the declaration of Helsinki.

## Results

### Socioeconomic characteristics of participants

The mean age of participants was 49.9 years (SD: 15.9). Majorities (57.7%) were females and 81 (15.5%) were born in non-European countries. Above half (56.2%) were married, whereas 62 (12.1%) attended less than 10 years of education.

### Item descriptives

Almost all items had sufficient variation to differentiate respondents’ level of knowledge. The correlation matrix indicated that a strong correlation between item 1 (‘*Stress is one of the main causes of heart attacks and strokes*’) and 5 (‘*Managing one’s stress levels will help to manage blood pressure*’) (Spearman’s rho (rs) = 0.76), and item 2 (‘*Walking and gardening are considered types of exercise that can lower the risk of having a heart attack or stroke*’) and 3 (‘*Moderately intense activity of 2½ hours a week will reduce your chances of having a heart attack or stroke.’*) (rs = 0.72). Similarly, all response options of the risk scale were well-represented except for item 4 (‘*The chance that I will have a heart attack or stroke within the next ten years is high*’) and 5 (‘*I will probably have a heart attack or stroke because of my past and/or current lifestyle behavior*’) with below 10 responses in the ‘*strongly agree*’ category. The correlation matrix indicated that strong correlation between item 16 (‘*I am considering eating at least 5 servings of fruit and vegetables a day*’) and reverse-coded item 20 (’*I am not thinking eating at least 5 servings of fruit and vegetables a day*’) (rs = 0.84), and item 10 (‘*I am considering exercising 30 min for at least 5 times a week’*) and reverse-coded item 13 (‘*I am not thinking exercising 30 min for at least 5 times a week*’) (rs = 0.86). Details on the frequencies of individual response options and correlation plots are available in the supplementary material.

### Exploratory and confirmatory factor analysis of the risk scale

**Figure 1:**
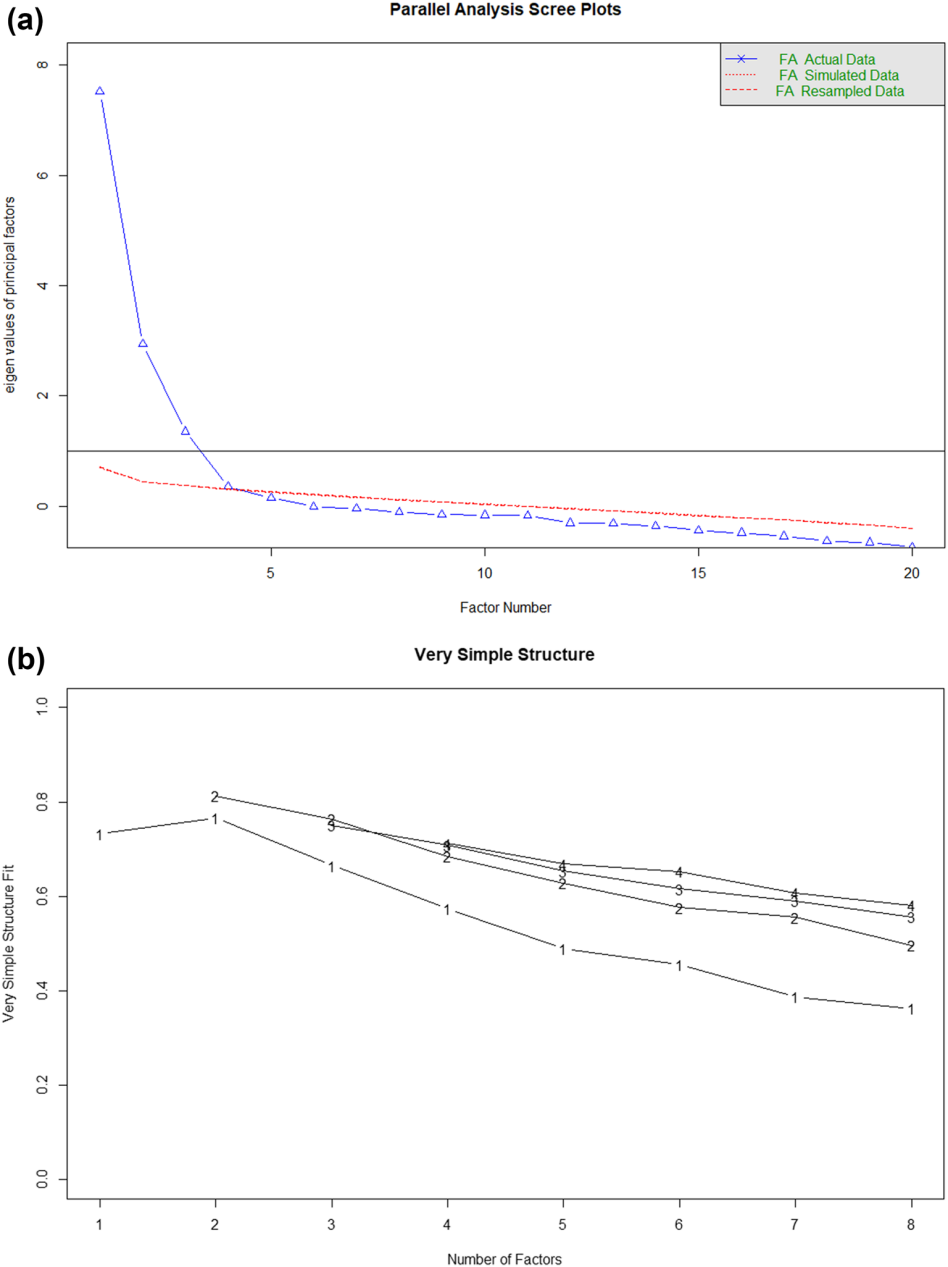
Scree plot (**a**) and very simple structure (**b**) of the exploratory factor analysis of the risk scale.

Our analysis demonstrated that the data was adequate for factor analysis based on the KMO (0.88) and Bartlett’s test of sphericity (2699.8, *p* < 0.001). Using the scree plot (Fig. 1a), and parallel analysis, a three-factor solution emerged, which accounted for 68.5% of the total variance. The very simple structure (Fig. 1b) also showed a three-factor solution was optimal (Velicer’s Minimum Average Partial test = 0.02).The pattern of factor loadings in our analysis slightly varied from the original subscales. The domains in the original subscales were risk perception, benefit finding and healthy eating intentions. Whereas in our analysis the domains are somehow similar with different loading patterns and we named them as; factor1—risk perception, factor2- perceived benefit and intention to change PA, and factor3—perceived benefit and intention to change dietary habit (Table 1). We performed a CFA and the results indicated that the model fit was moderately acceptable (X^2^ = 560.7, df = 167, *p* < 0.001; CFI = 0.92; TLI = 0.91; RMSEA = 0.07, 95% confidence interval (CI) (0.06, 0.8); SRMR = 0.04). Standardized covariances ranged from 0.10 (*p* < 0.01) between factor 1 and 2 to 0.25 (*p* < 0.001) between factor 2 and 3. Extended results of the CFA are available in the supplementary material.

**Table 1:** Factor loadings of the exploratory factor analysis of the ABCD risk scale.

*Item no*	Factor 1	Factor 2	Factor 3	Communality	Uniqueness
1	**0.72**	0.07	− 0.13	0.51	0.49
2	**0.74**	0.02	− 0.03	0.59	0.41
3	**0.64**	− 0.004	0.23	0.47	0.53
4	**0.78**	− 0.06	0.09	0.63	0.37
5	**0.66**	0.02	0.14	0.47	0.53
6	** − 0.69**	0.02	0.05	0.50	0.50
7	**0.78**	0.01	− 0.05	0.59	0.41
8	0.12	**0.71**	− 0.09	0.51	0.49
9	0.10	**0.83**	− 0.02	0.73	0.27
10	− 0.03	**0.72**	0.05	0.52	0.48
11	− 0.05	**0.77**	0.14	0.71	0.29
12	0.07	**0.35**	0.19	0.31	0.69
13	0.05	** − 0.78**	0.06	0.64	0.36
14	− 0.04	0.21	**0.61**	0.55	0.45
15	0.02	0.25	**0.78**	0.65	0.35
16	0.04	0.07	**0.77**	0.54	0.46
17	0.02	0.02	**0.69**	0.50	0.50
18	0.10	− 0.08	**0.71**	0.51	0.49
19	− 0.03	0.19	**0.51**	0.39	0.61
20	0.04	0.03	** − 0.79**	0.64	0.36

The applied rotation method was **oblimin**. Factor loadings with absolute values higher than 0.3 are in bold; Factor 1: Risk perception; Factor 2: Perceived benefit and intention to change PA; Factor 3: Perceived benefit and intention to change dietary habit.

### Mokken scaling analysis

The MSA showed that the coefficient of homogeneity (H) for the whole knowledge scale was 0.38 (standard error (SE) = 0.03), which is a weak but acceptable scalability. However, individual item coefficients showed that items 6 (‘*Drinking large amounts of alcohol can increase your cholesterol and other blood fats*’) and 7 (‘*There is 'good' and there is 'bad' cholesterol*’) had H below 0.3, which violates the criterion of scalability. Furthermore, these items also showed deviation from monotonicity.

The H for the entire risk scale was 0.44 (SE = 0.03), indicating a moderate accuracy. However, the individual H values and monotonicity indices indicated violation of homogeneity and monotonicity for some items. Then, we performed the MSA on each subscale of the risk scale separately based on the results of EFA. Hence, the overall H was 0.64 (SE = 0.02), 0.71 (SE = 0.02) and 0.72 (SE = 0.02) for risk perception, perceived benefit of PA, and dietary habit, respectively. Moreover, all items in the respective subscales had H positive and above 0.3, which is an acceptable level of homogeneity. Details of the MSA of the knowledge scale and each domain of the risk scale are available in the supplementary material.

### Rasch and two-parametric logistic (2-PL) model of the knowledge scale

**Figure 2:**
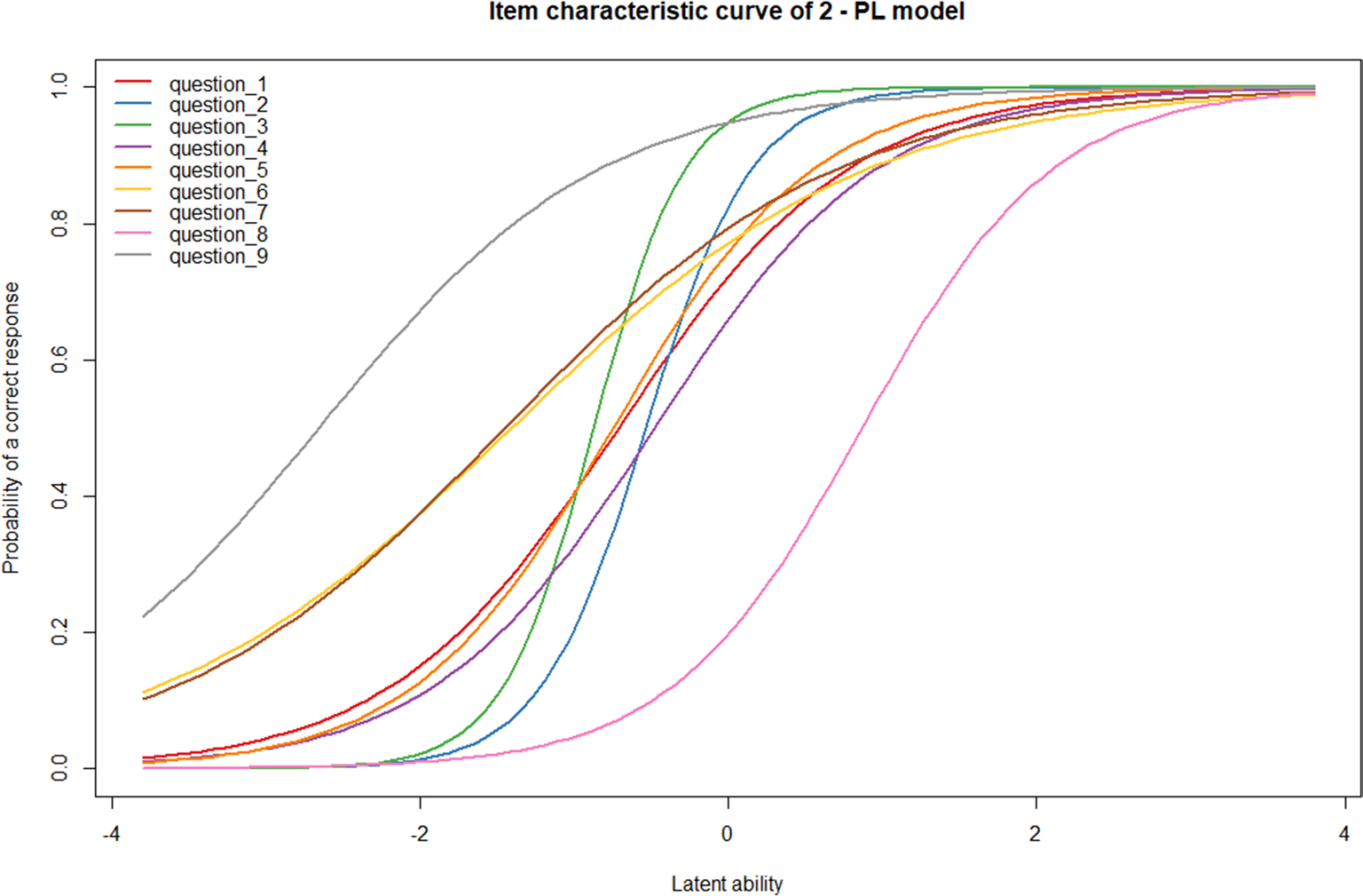
The joint Item Characteristic Curves (ICCs) of the two—parametric logistic model of ABCD-knowledge scale.

**Figure 3:**
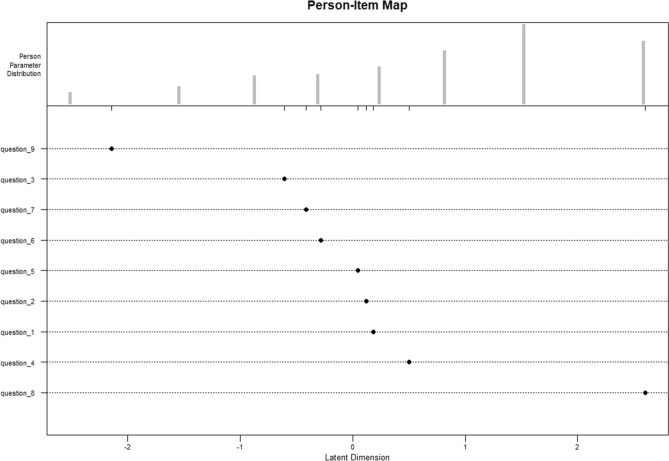
The person-item map of the Rasch model of ABCD-knowledge scale.

We compared the performance of the Rasch and 2-PL model using the Likelihood-Ratio (LR) test and the fit was significantly different (*p* < 0.001), indicating that the more complex 2-PL model performed better than the Rasch model. Hence, the results of the 2-PL model are summarized in Table 2 and the ICCs are displayed in Fig. 2. The result showed that question 9 was the easiest (%Correct = 92.0%; δ = − 2.65; SE = 0.41) and question 8 was the most difficult item (%Correct = 27.8%; δ = 0.87; SE = 0.11). The discrimination parameters also showed heterogeneity, question 3 and 6 respectively had the highest (α = 3.82; SE 0.77) and the lowest (α = 0.86; SE = 0.14) discrimination. The model fit was adequate, with average infit and outfit mean square statistics (MSQ) around 1.00. The infit and outfit MSQs for all individual items were within the acceptable range, except for item 3, in which the infit and outfit value was 0.34 and 0.49 (below 0.6). The person-item map showed that item saturation around average levels of the latent trait and beyond ± 2, and item deficiency in between ± 1 to ± 2 (Fig. 3).

**Table 2:** Summary of 2—PL model parameters and item fit statistics of the ABCD knowledge scale.

Item	% Correct	Delta (SE)	Alpha (SE)	Outfit		Infit	
				MSQ	P	MSQ	P
1	67.2	− 0.72 (0.11)	1.31 (0.20)	0.76	0.007	0.96	0.510
2	68.4	− 0.52 (0.07)	3.20 (0.60)	0.66	0.002	0.60	0.000
3	78.3	− 0.84 (0.08)	3.82 (0.77)	0.34	0.000	0.49	0.000
4	62.1	− 0.47 (0.09)	1.42 (0.20)	0.98	0.849	1.01	0.876
5	69.3	− 0.75 (0.11)	1.50 (0.22)	0.75	0.008	0.93	0.307
6	74.1	− 1.40 (0.22)	0.86 (0.14)	1.34	0.016	1.33	0.000
7	76.0	− 1.43 (0.21)	0.94 (0.15)	1.42	0.004	1.32	0.000
8	27.8	0.87 (0.11)	1.58 (0.25)	0.70	0.569	0.92	0.698
9	92.0	− 2.65 (0.41)	1.09 (0.22)	0.56	0.004	0.78	0.000

2—PL: Two-parametric logistic model; MSQ: Mean-square; SE: standard error; Delta (δ): Difficulty; Alpha (α): Discrimination.

The estimated difficulty parameters of the Rasch model are roughly comparable to those from the 2-PL model with strong correlation in between (Pearson correlation coefficient (r) = 0.94). Details of the Rasch estimates, ICCs and LR parameters are provided in the supplementary material.

### Rasch Rating Scale Model (RSM) of the risk scale

Table 3 presents the summary of item parameter estimates and infit and outfit statistics for each domain of the risk scale. We investigated whether τi1 ≤ τi2, and τi2 ≤ τi3 for each item and the result indicates the thresholds were ordered as expected given the ordinal rating scale categories for all items in all three subscales. Moreover, infit and outfit values for all the items were between 0.6 and 1.4, except for items 11 and 16 which fall slightly below 0.6, indicating that these items had less variation in the observed response pattern than expected by the model. Further details of individual ICCs, person-item maps for risk scale are available in the supplementary material.

**Table 3:** Summary of parameter estimates and item fit statistics of the Rating Scale Model: ABCD—risk scale.

Item	Mean	Severity (SE)	Outfit	Infit	τi.Cat1	τi .Cat2	τi .Cat3
Risk perception							
1	1.20	1.12 (0.09)	1.04	1.06	− 2.65	1.14	4.88
2	1.13	1.42 (0.09)	0.86	1.88	− 2.36	1.44	5.18
3	1.22	1.06 (0.09)	1.14	1.16	− 2.72	1.07	4.82
4	0.97	2.05 (0.09)	0.79	0.79	− 1.72	2.07	5.81
5	0.95	2.15 (0.09)	0.89	0.90	− 1.63	2.16	5.91
6	1.13	1.42 (0.09)	1.11	1.09	− 2.36	1.43	5.18
7	1.04	1.78 (0.09)	0.91	0.92	− 2.00	1.79	5.54
Perceived benefit and intention to change of PA							
8	2.11	− 2.03 (0.09)	1.28	1.13	− 5.10	− 2.31	1.31
9	2.08	− 1.92 (0.09)	0.90	0.92	− 4.98	− 2.20	1.43
10	1.87	− 1.13 (0.09)	0.62	0.66	− 4.20	− 1.41	2.21
11	1.89	− 1.20 (0.09)	0.59	0.63	− 4.27	− 1.49	2.14
12	1.69	− 0.49 (0.08)	1.24	1.24	− 3.56	− 0.77	2.85
13	1.91	− 1.26 (0.09)	0.99	1.01	− 4.32	− 1.54	2.09
Perceived benefit and intention to change of dietary habit							
14	1.92	− 1.55 (0.09)	1.16	1.12	− 5.21	− 1.65	2.21
15	1.80	− 1.06 (0.09)	0.82	0.83	− 4.72	− 1.17	2.69
16	1.68	− 0.58 (0.09)	0.52	0.54	− 4.24	− 0.69	3.17
17	1.72	− 0.74 (0.09)	0.60	0.62	− 4.39	− 0.84	3.02
18	1.60	− 0.28 (0.09)	0.87	0.88	− 3.93	− 0.38	3.48
19	1.65	− 0.46 (0.09)	1.36	1.36	− 4.11	− 0.56	3.30
20	1.74	− 0.82 (0.09)	1.08	1.10	− 4.48	− 0.92	2.94

τi = Threshold of respective categories.

### Reliability analysis

The knowledge scale showed acceptable internal consistency with Cronbach’s alpha of 0.75; Guttman’s lambda-6 = 0.90; Beta = 0.58; and Omega = 0.74 (95% CI 0.70–0.78). The reliability statistics if item deleted showed no major change except for minimal increase in Cronbach’s alpha from 0.75 to 0.77 when item 7 was dropped. We computed reliability indices for each of the subscales of the risk scale and the Cronbach’s alpha coefficients were 0.93, 0.88 and 0.84 for risk perception, intention to change PA, and dietary habit, respectively, indicating an excellent internal consistency. Similarly, no major changes were observed in the reliability statistics when items deleted. Details of the reliability statistics for both knowledge and each domain of the risk scale are available in the supplementary material.

### Short scale version

We also tested the reliability of a short version of ABCD questionnaire. Based on the factor loadings, psychometric properties (CTT and IRT), and subjective judgement, we selected seven items from the knowledge scale, and five items from each construct of the risk scale. The shorter version of the knowledge scale (7-items) provided a good internal consistency (Cronbach’s alpha = 0.76; Guttman’s lambda-6 = 0.79; Beta = 0.54; and Omega = 0.71 (95% CI 0.65, 0.78)). The correlation with the original version was sufficient (r = 0.95). Similarly, the shorter version of risk perception, perceived benefit of PA and dietary habit showed an excellent internal consistency (Cronbach’s alphas equal to 0.91, 0.85, and 0.82, respectively) with strong correlation with their original versions. Details are available in the supplementary material.

## Discussion

With the continuing burden of CVDs, targeting knowledge and risk perception of adults is vital to improve healthy lifestyle. In response to the need for a valid and reliable tool to measure CVD knowledge and risk perception, Woringer and her colleagues developed the ABCD questionnaire. To the best of our knowledge, the ABCD questionnaire is not translated and validated to be used in Belgium. Therefore, we conducted a validation study on a random sample of adults in selected districts to examine the psychometric properties of the Dutch (Flemish) version of the questionnaire. Our analyses demonstrated that both the knowledge and each domain of the risk scale have good psychometric properties. Hence, the Flemish version of ABCD questionnaire could be a valuable tool for researchers, public health and clinical practitioners to assess an individual’s level of CVD knowledge, self-perceived risk to take appropriate measures accordingly. Furthermore, it can be utilized to monitor and evaluate the effectiveness of interventions and programs aimed at improving CVD risks awareness, perception and intention to change. For coaching and intervention purposes specific items might be considered as some items more emphasize on the perceived benefit, whereas some others focus on intention to change and self-efficacy measures.

We confirmed a three-factor structure for the 20-item risk scale comprising risk perception, perceived benefit and intention to change PA, and perceived benefit and intention to change dietary habits. These three factors accounted for 68.5% of the total variance. The pattern of factor loadings in our analysis was slightly different from the original subscales. The original subscales were risk perception, benefit finding and healthy eating intentions. The Hungarian version of ABCD questionnaire also replicated the same constructs as the original ones, however, the pattern of factor loadings somewhat varied^33^. In our analysis the factor loading of the two latent constructs are slightly different from the original subscales. Item 14 (‘*When I eat at least five portions of fruit and vegetables a day I do something good for the health of my heart.*’), which was in factor 2 showed a better loading to factor 3 in our analysis. The two additional items related to healthy dietary habit also loaded to factor 3 along with three items of the original subscale (health eating intention). We tested the factorial structure using CFA and the model fit was better. The two added items have similar construct with the 3 items of healthy eating intentions (factor-3) of the original subscales and item 14 which is logically related to healthy diet intention rather than PA. Furthermore, the difference in ordering of the questions could also contribute to the variation in factorial structure^34,35^.

As demonstrated in the MSA, most of the items in the knowledge scale have good scalability and monotonicity coefficients, except for items 6 and 7, which might not be applicable as a unidimensional scale with other items. This implies that these items might have a different underlying construct compared to the rest of the items. Hence, using these items along with other items to measure CVD knowledge might be misleading. Whereas, the rest of the knowledge scale items are homogenous and unidimensional. Likewise, each domain of the risk scale showed a good scalability property, indicating that the subscales are sufficiently homogenous and the application of risk subscales by adding the individual item scores could be suitable to measure underlying constructs.

The 2-PL model showed that the infit and outfit statistics of most of the knowledge items were within the acceptable range, implying that the items have sufficient contribution to the overall score of knowledge. Furthermore, the model also showed that items differ not only in difficulty but also in discriminatory potential. However, items of the knowledge scale are saturated around the average levels of knowledge, indicating the need for additional items to better locate individuals’ CVD risk knowledge level. In this regard, the Rasch model also indicated on average, the respondents were located higher than the knowledge scale, suggesting that the items might be relatively easy for participants. Thus, adding more difficult items of various dimensions on the knowledge scale is recommended to provide a better insight in the individual’s CVD knowledge level and to differentiate participants sufficiently in this regard.

The Rasch rating scale model for each domain of the risk scale showed that infit and outfit statistics for all the items were within the acceptable range, except for item 11 and 16, which fall below 0.6, showing items had less variation in the observed response pattern and might have strong correlation with other items. This signifies the possibility of item modification or reduction in the risk scale.

Our reliability analysis indicated that the knowledge scale has moderate internal consistency with Cronbach’s alpha of 0.75, which is higher than the result shown in the previous validation study in Hungary and Australia^33,36^. The reliability indices for each subscales of the risk scale showed a good internal consistency, which is coherent with the findings of the original study, the Hungarian version and a revalidation in Australia, though the loadings vary to some extent^22,33,36^.

We reduced the items and tested the psychometric properties of a short version of the ABCD questionnaire. The shorter version of the knowledge scale (7-item) is almost as good as the original one based on the internal consistency measures and it also has sufficient correlation with the original scale. However, internal consistency measures might not always be a good indicator as smaller number of items usually resulted in a better consistency. Moreover, as indicated in the Rasch analysis, the knowledge items are relatively easy and not sufficient at each level of participants’ latent ability. Hence, we recommend changing item 6 and 7 as well as adding more items of the knowledge in order to measure multiple dimensions of CVD knowledge. The shorter version of risk perception, perceived benefit and intention to change of physical activity, and diet showed an excellent internal consistency and strong correlation with the scores of respective longer versions. Therefore, this study showed that the ABCD questionnaire could be applicable in future studies, clinical and public health practices to measure an individual’s CVD knowledge and risk perception.

The present study has limitations that need to be considered in interpretation of findings. First, due to the nature of postal and online surveys, we had a high rate of non-response. However, the use of matched substitution technique helped the survey to have a prefixed net sample sizes within age groups, sex and geographical location that improved the representativeness across socioeconomic groups. Second, we did not measure other variables such as family history of CVD and self-perceived health, which might theoretically be related to dietary and physical activity behaviors.

In conclusion, results of the psychometric investigation suggest that the Flemish version of ABCD risk questionnaire has an acceptable property to assess an individual’s CVD risk perception, perceived benefit and intention to change physical activity and dietary habit. Hence, the measure is acceptable to be used among adults in Belgium, particularly Flanders context. Moreover, with minor contextual modifications, the questionnaire could be employed in the Netherlands and other areas where Dutch-speaking communities live. Similarly, the ABCD knowledge scale also showed good psychometric property, however, the item was relatively easy and it might not be comprehensive to assess many aspects of CVD risk factors. Therefore, we recommend addition of more items in order to have a better insight on knowledge and to be able to differentiate participants well. Since we did not use a French or German version of the questionnaire, the results might not be generalizable to French- and German-speaking communities within Belgium.

## Supplementary Information

Supplementary Information

## Data Availability

Data will be available on reasonable request.
