# Supplementary material for: Validation of the Dutch-Flemish translated ABCD questionnaire to measure cardiovascular diseases knowledge and risk perception among adults
Source: Sci Rep. 2021 Apr 26;11:8952. doi: 10.1038/s41598-021-88456-5 (PMC8076268; doi:10.1038/s41598-021-88456-5)
Supplement: Supplementary file 1 — Supplementary Information [file 41598_2021_88456_MOESM1_ESM.pdf]

Supplementary materials: Validation of the Dutch-Flemish translated ABCD questionnaire to measure cardiovascular diseases knowledge and risk perception among adults

Hamid Yimam Hassen[1]*, Naomi Aerts[1], Stefaan Demarest[4], Md Dilshad Manzar[5], Steven Abrams[2,3¥], and Hilde Bastiaens[1,2¥]

[1] Department of Primary and Interdisciplinary Care, Faculty of Medicine and Health Sciences, University of Antwerp, Antwerp, Belgium

[2] Global Health Institute, Faculty of Medicine and Health Sciences, University of Antwerp, Antwerp, Belgium

[3] Interuniversity Institute for Biostatistics and statistical Bioinformatics, Data Science Institute, Hasselt University, Diepenbeek, Belgium

[4] Department of Public Health and Surveillance, Scientific Institute of Public Health, 14, Juliette Wytsmanstraat, 1050, Brussels, Belgium

[5] Department of Nursing, College of Applied Medical Sciences, Majmaah University, Al Majmaah, Saudi Arabia

*Correspondence to:

Hamid Yimam Hassen,

Email: Hamid.hassen@uantwerpen.be

Telephone: +32466298748

¥ - These authors share senior co-authorship.

Contents

Contents ... 2
Item frequencies ... 3
  knowledge scale ... 3
  Risk scale ... 4
Inter-item correlations ... 5
  knowledge scale ... 5
  Risk scale ... 5
Mokken Scaling Analysis (MSA) ... 7
Factor analysis ... 8
  Exploratory factor analysis ... 8
  Confirmatory factor analysis ... 10
Item properties - parametric Item Response Theory ... 12
  Rasch and 2 – PL model: knowledge scale ... 12
  Comparing Rasch and 2-PL models ... 13
Rating Scale Model – Risk scale ... 14
Classical Test Theory analysis ... 17
Reliability statistics for the shorter form ... 17
Dutch (Flemish) version of the ABCD questionnaire ... 18

Item frequencies

knowledge scale

The tendency of endorsing correct answers in the individual knowledge questions are presented in table S1. The results show that almost all items have sufficient variation to differentiate respondents. The differences between items in response frequencies indicates the difference in the item difficulty. One hundred forty-six (27.8%) of participants know family history of CVD as a risk factor, while almost all (92.0%) know that smoking is a risk factor for CVD.

Table S1. Item response frequencies for the 9-item knowledge scale (n=525)

**Knowledge questions**	**Responded correctly**	
	**Frequency**	**(%)**
1. Stress is one of the main causes of heart attacks and strokes.	353	67.2
2. Walking and gardening are considered types of exercise that can lower the risk of having a heart attack or stroke.	359	68.4
3. Moderately intense activity of 2 ½ hours a week will reduce your chances of having a heart attack or stroke.	411	78.3
4. People who have diabetes are at higher risk of having a heart attack or stroke	326	62.1
5. Managing your stress levels will help you to manage your blood pressure	364	69.3
6. Drinking large amounts of alcohol can increase your cholesterol and other blood fats	389	74.1
7. There is 'good' cholesterol and there is 'bad' cholesterol.	399	76.0
8. A family history of heart disease is not a risk factor for high blood pressure	146	27.8
9. People who smoke have an increased risk of cardiovascular disease	483	92.0

Risk scale

The frequency and percentage of endorsing individual response options of the risk scale are presented in table S2 below. Almost all items have sufficient variation to differentiate respondents except for item 4 and 5 with below 10 responses in the "*strongly agree*" option.

Table S2. Frequency and percent of item response options for the 20-item risk scale (n=525)

**Risk**	**Strongly disagree**	**Disagree**	**Agree**	**Strongly agree**
1. I have the feeling that someday I will suffer a heart attack or stroke in my life.	86 (16.4)	265 (50.5)	147 (28.0)	22 (4.2)
2. It is likely that I will have a heart attack or stroke in the future.	95 (18.1)	278 (53.0)	133 (25.3)	14 (2.7)
3. It is possible that I will have a heart attack or stroke within the next ten years.	96 (18.3)	225 (43.0)	192 (36.6)	10 (1.9)
4. The chance that I will have a heart attack or stroke within the next ten years is high.	124 (23.6)	297 (56.6)	91 (17.4)	9 (1.7)
5. I will probably have a heart attack or stroke because of my past and/or current lifestyle behavior.	135 (25.7)	286 (54.5)	96 (18.3)	6 (1.1)
6. I am not worried that I might have a heart attack or stroke.	103 (19.6)	276 (52.6)	122 (23.2)	24 (4.6)
7. I am concerned because there is a chance that I will have a heart attack or stroke in the near future.	122 (23.2)	269 (51.2)	113 (21.5)	15 (2.9)
8. When I exercise for 30 minutes at least 5 times a week, I do something good for the health of my heart.	22 (4.2)	50 (9.5)	303 (57.7)	149 (28.4)
9. It is important for me to exercise regularly.	28 (5.3)	77 (14.7)	246 (46.9)	173 (33.0)
10. I am considering exercising 30 minutes for at least 5 times a week.	31 (5.9)	120 (22.9)	250 (47.6)	115 (21.9)
11. I intend or want to exercise 30 minutes for at least 5 times a week.	29 (5.5)	112 (21.3)	263 (50.1)	112 (21.3)
12. I am sure that I can gain or maintain a healthy weight by exercising 30 minutes for at least 5 times a week in the next 2 months.	36 (6.9)	164 (31.2)	246 (46.9)	76 (14.5)
13. I am not considering exercising 30 minutes for at least 5 times a week.	31 (5.9)	127 (24.2)	219 (41.7)	140 (26.7)
14. When I eat at least 5 servings of fruit and vegetables a day, I do something good for the health of my heart	30 (5.7)	93 (17.7)	291 (55.4)	109 (20.8)
15. It is important for me to eat at least 5 servings of fruit and vegetables every day.	26 (5.0)	140 (26.7)	271 (51.6)	86 (16.4)
16. I am considering eating at least 5 servings of fruit and vegetables a day.	29 (5.5)	179 (34.1)	245 (46.7)	69 (13.1)
17. I intend, or want, to eat at least 5 servings of fruit and vegetables a day.	32 (6.1)	157 (29.9)	259 (49.3)	74 (14.1)
18. I am sure that I can eat at least 5 servings of fruit and vegetables a day for the next 2 months.	42 (8.0)	197 (37.5)	212 (40.4)	72 (13.7)
19. I am sure that I can gain or maintain a healthy weight by eating at least 5 servings of fruit and vegetables a day in the next 2 months.	40 (7.6)	183 (34.9)	224 (42.7)	77 (14.7)
20. I am not considering eating at least 5 servings of fruit and vegetables a day.	45 (8.6)	164 (31.2)	196 (37.3)	117 (22.3)

Inter-item correlations

knowledge scale

The correlation matrix indicate that strong correlation exist between item 1 and 5 (rs=0.76), item 2 and 3 (rs=0.72). Moderate correlation was observed between item 3 and 4 (rs=0.59) and item 2 and 4 (rs=0.42). Items 7 shows consistent weak associations with other items. (figure S1)

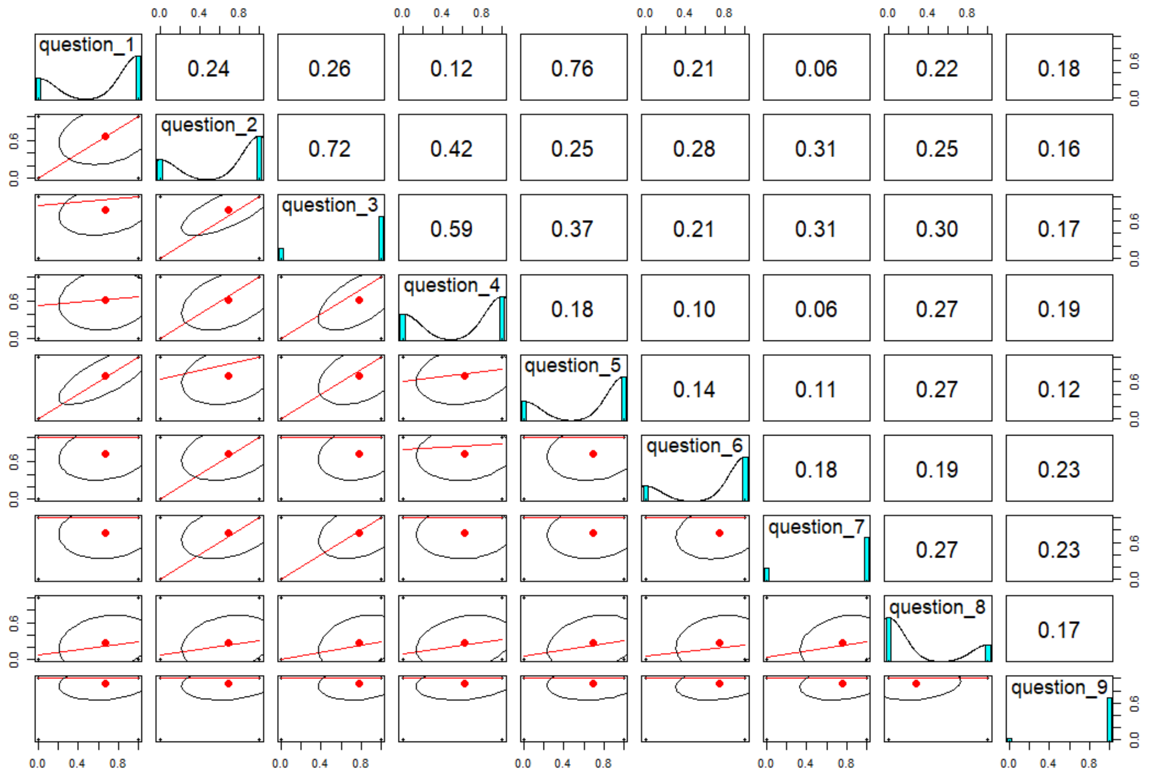

Figure S1. Knowledge scale (9 items): Correlation-plot of Spearman correlations between item scores

Risk scale

The correlation matrix indicate that strong correlation exists between item 16 and 20 (reverse coded) (rs=0.87), item 16 and 17 (rs=0.84), and item 10 with 11 (rs=0.84) and 13 (reverse coded) (rs=0.84). Similarly, moderate correlation exist between item 1 and 2 (rs=0.67), 11 and 13 (rs=0.74), 15 and 16 (rs=71), item 6 and 7 (rs=0.75), 16 and 18 (rs=0.74). No negative correlation was observed between items. (figure S2)

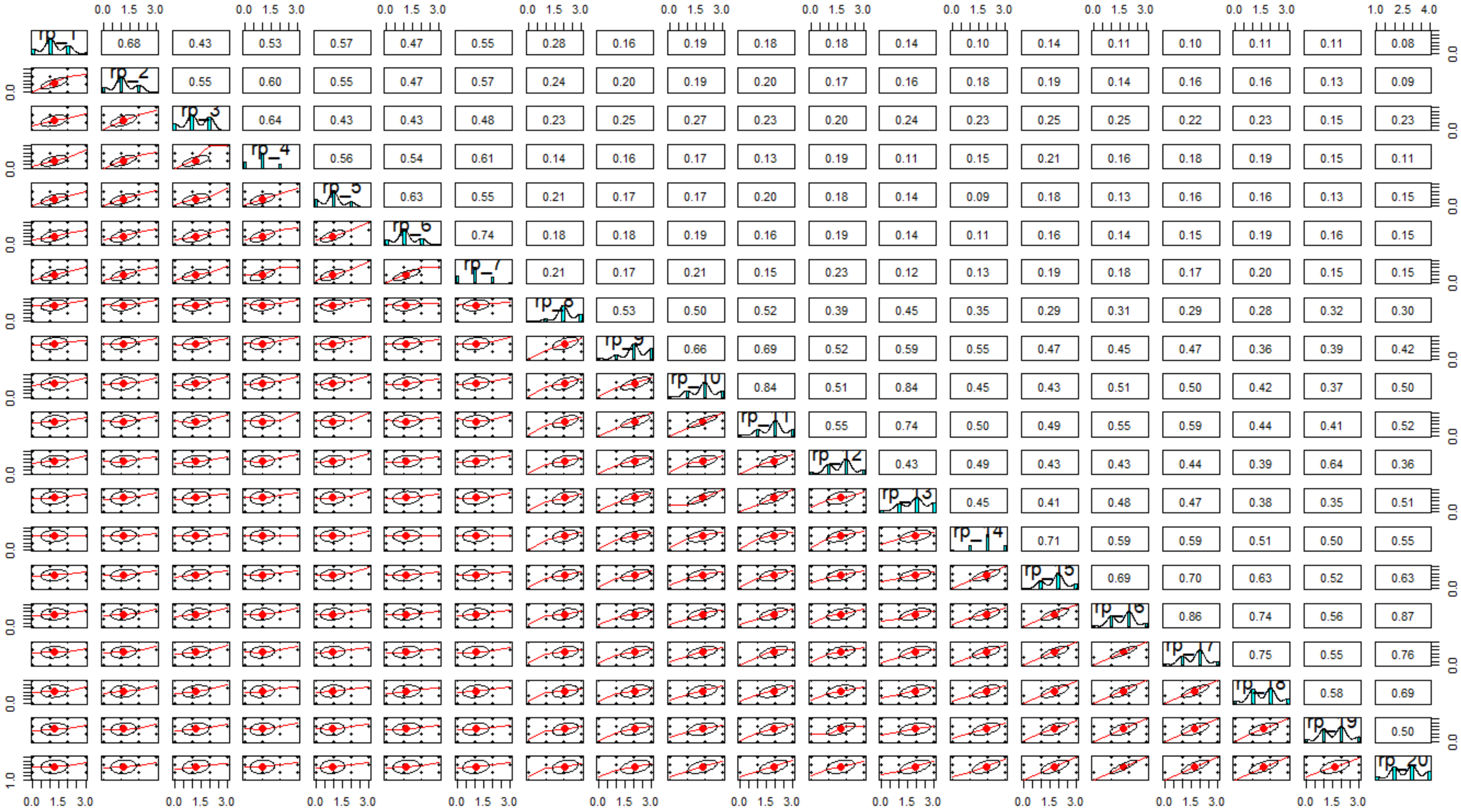

Figure S2. Risk scale (20-items): Correlation-plot of Spearman correlations between item scores

Mokken Scaling Analysis (MSA)

The output shows that all Hjs are positive satisfying the first criterion of a Mokken scale. Items 6 (Hj=0.246) and 7 (Hj=0.244) have Hjs less than 0.3, which violates the second criterion of a Mokken scale. The scalability coefficient for the entire scale, H, equals 0.376 (se=0.025), which is weak but acceptable. (table S3)

Table S3. MSA: knowledge scale: item salability and monotonicity (n=523 complete cases)

Items	H (SE)	#ac	#vi	#vi/#ac	maxvi	sum	sum/#ac	zmax	#zsig	crit
1	0.340 (0.032)	10	0	0	0	0	0	0	0	0
2	0.425 (0.028)	15	0	0	0	0	0	0	0	0
3	0.531 (0.027)	10	0	0	0	0	0	0	0	0
4	0.337 (0.034)	15	0	0	0	0	0	0	0	0
5	0.364 (0.030)	6	0	0	0	0	0	0	0	0
6	0.246 (0.035)	15	1	0.07	0.03	0.03	0.002	0.44	0	19
7	0.244 (0.038)	15	2	0.13	0.04	0.09	0.006	0.6	0	33
8	0.647 (0.038)	6	0	0	0	0	0	0	0	0
9	0.422 (0.060)	6	0	0	0	0	0	0	0	0

H(SE): scalability coefficient with standard error; #Ac: active pairs; #vi: violations; maxvi: maximum violations; sum : sum of violations of manifest monotonicity; sum/#ac: average violation per active pair;

The MSA for risk scale shows all items have Hi positive and above 0.3, showing a good scalability in the scale. (table S4)

Table S4. MSA: Risk scale: item scalability and monotonicity (n=494 complete cases)

Items	H (SE)	#ac	#vi	#vi/#ac	maxvi	Sum	sum/#ac	zmax	#zsig	crit
**Risk perception (overall H = 0.635 (0.02))**										
1	0.61 (0.03)	35	1	0.03	0.06	0.06	0.0016	0.97	0	2
2	0.64 (0.02)	33	0	0	0	0	0	0	0	0
3	0.60 (0.03)	26	0	0	0	0	0	0	0	0
4	0.68 (0.03)	25	0	0	0	0	0	0	0	0
5	0.65 (0.03)	30	1	0.03	0.03	0.03	0.0011	0.29	0	-6
6	0.61 (0.02)	21	1	0.05	0.05	0.05	0.0024	0.88	0	4
7	0.65 (0.02)	24	0	0	0	0	0	0	0	0
**Perceived benefit and intention to change of PA (overall H = 0.71 (0.02))**										
8	0.64 (0.04)	30	0	0	0	0	0	0	0	0
9	0.75 (0.02)	31	0	0	0	0	0	0	0	0
10	0.77 (0.02)	24	0	0	0	0	0	0	0	0
11	0.78 (0.02)	33	0	0	0	0	0	0	0	0
12	0.62 (0.03)	40	1	0.03	0.03	0.03	0.0008	0.9	0	-3
13	0.7 (0.02)	31	0	0	0	0	0	0	0	0
**Perceived benefit and intention to change of healthy dietary habit (overall H = 0.725 (0.02))**										
14	0.69 (0.03)	37	1	0.03	0.07	0.07	0.0019	1.16	0	1
15	0.73 (0.02)	28	0	0	0	0	0	0	0	0
16	0.79 (0.02)	13	1	0.08	0.03	0.03	0.0027	0.68	0	-5
17	0.78 (0.02)	18	0	0	0	0	0	0	0	0
18	0.74 (0.02)	49	2	0.04	0.18	0.22	0.0045	2.93	1	37
19	0.60 (0.03)	40	1	0.03	0.08	0.08	0.0019	1.06	0	6
20	0.74 (0.02)	31	0	0	0	0	0	0	0	0

H(SE): scalability coefficient with standard error; #Ac: active pairs; #vi: violations; maxvi: maximum violations; sum : sum of violations of manifest monotonicity; sum/#ac: average violation per active pair;

Factor analysis

Exploratory factor analysis

we performed an exploratory (EFA) using the maximum likelihood extraction and Oblimin rotation method on random half of the sample (n=262). We evaluated the sample and data adequacy for factor analysis using Kaiser-Meyer-Olkin (KMO) and Bartlett's test of sphericity. KMO values between 0.8 and 1 indicate the sampling is adequate, whereas values less than 0.6 show the sampling is not adequate. We determined the adequate number of factors using a scree plot and parallel analysis, which was based on the eigenvalues of randomly generated multiple data matrices that have equal number of items and sample as the original dataset. Detailed results are available below.

Statistics of the exploratory factor analysis

**Statistics**	**MR2**	**MR3**	**MR1**
SS loadings	3.76	3.72	3.36
Proportion Var	0.19	0.19	0.17
Cumulative Var	0.19	0.37	0.54
Proportion Explained	0.35	0.34	0.31
Cumulative Proportion	0.35	0.69	1.00

**Statistics**	**Values**
Tucker Lewis Index of factoring reliability	0.910
RMSEA index (90%CI)	0.092 (0.083, 0.102)
BIC	-309.83
RMSR	0.04

Measures of factor score adequacy

Measures of factor score adequacy	**Factor 2**	**Factor 3**	**Factor 1**
Correlation of (regression) scores with factors	0.94	0.95	0.95
Multiple R square of scores with factors	0.89	0.90	0.90
Minimum correlation of possible factor scores	0.78	0.79	0.80

With factor correlations

	MR1	MR2	MR3
MR1	1	0.24	0.36
MR2	0.24	1	0.53
MR3	0.36	0.54	1

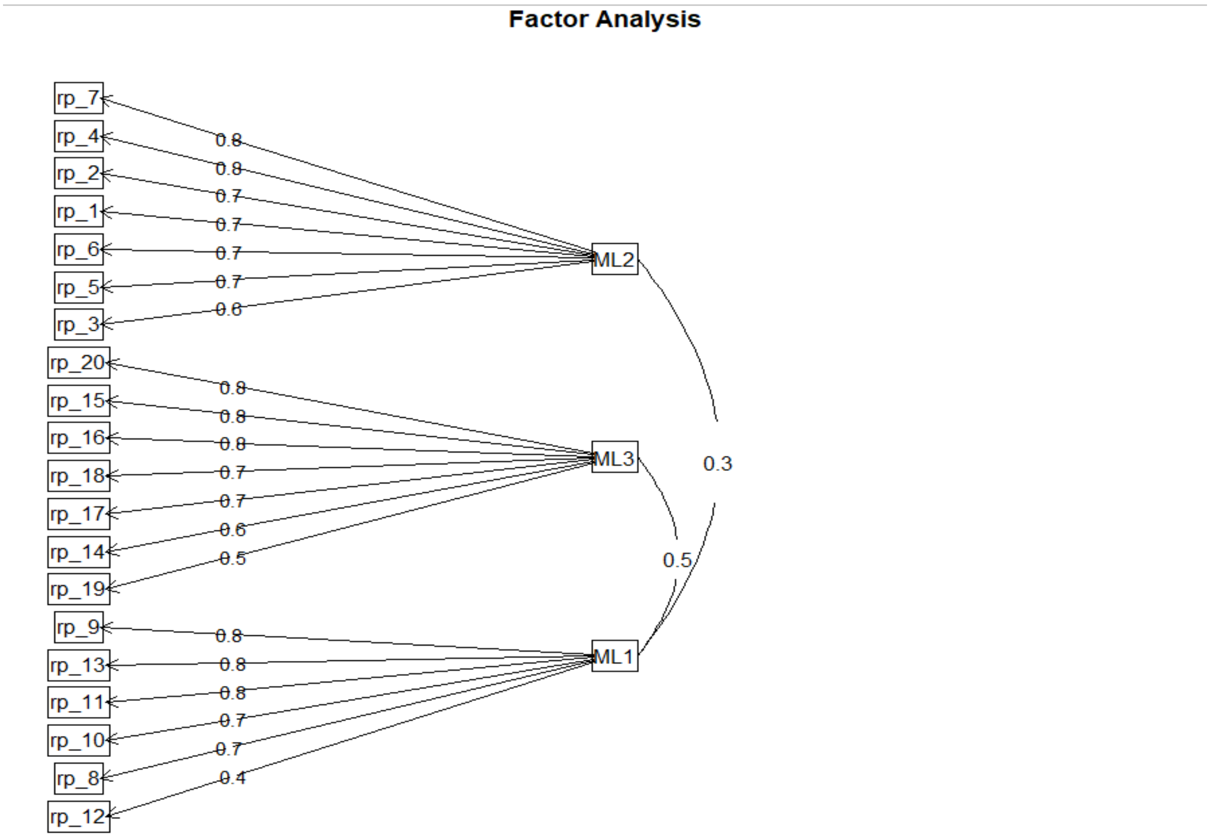

Figure S3. Factor structure of the exploratory factor analysis of the risk scale. (Cluster fit = 0.92, Pattern fit = 0.97, RMSR = 0.08)

Confirmatory factor analysis

We evaluated the model fit of the CFA using; the X^2 test, the Tucker-Lewis and Comparative Fit Indexes (TLI and CFI, respectively; values 0.90 to 0.95 indicate acceptable fit, while values > 0.95 suggest good fit) and the root mean square error of approximation (RMSEA; values < 0.08 indicate an acceptable fit, while values < 0.05 indicate a good fit).

Parameter estimates

Table S5. Parameter estimates of the confirmatory factor analysis of the risk scale

Latent variable:

Items	Estimate	SE	Z	p-value
Rp				
1	1	0	-	-
2	0.954	0.070	13.716	0
3	0.869	0.077	11.210	0
4	0.888	0.066	13.349	0
5	0.915	0.066	13.913	0
6	0.956	0.075	12.706	0
7	0.979	0.072	13.568	0
Pa				
8	1	0	NA	NA
9	1.389	0.130	10.660	0
10	1.416	0.137	10.361	0
11	1.476	0.129	11.433	0
12	0.991	0.113	8.756	0
13	1.404	0.129	10.877	0
Diet				
14	1	0	NA	NA
15	0.981	0.072	13.588	0
16	0.901	0.075	11.967	0
17	0.872	0.069	12.677	0
18	0.847	0.079	10.735	0
19	0.866	0.077	11.249	0
20	1.037	0.080	12.903	0

Covariances:

	Estimate	SE	Z	p-value
Rp~~				
Pa	0.101	0.024	4.199	0.000
Diet	0.118	0.031	3.849	0.000
Pa~~				
Diet	0.250	0.036	6.986	0.000

Variances:

Items	Estimate	SE	Z	P-value
1	0.239	0.025	9.459	0
2	0.189	0.020	9.209	0
3	0.331	0.032	10.197	0
4	0.184	0.020	9.419	0
5	0.162	0.018	9.080	0
6	0.260	0.027	9.719	0
7	0.209	0.022	9.298	0
8	0.330	0.032	10.352	0
9	0.262	0.028	9.308	0
10	0.329	0.034	9.615	0
11	0.156	0.020	7.711	0
12	0.359	0.034	10.423	0
13	0.230	0.026	9.013	0
14	0.279	0.029	9.465	0
15	0.208	0.023	8.999	0
16	0.298	0.030	9.855	0
17	0.224	0.023	9.552	0
18	0.380	0.037	10.229	0
19	0.341	0.034	10.091	0
20	0.295	0.031	9.435	0
RP	*0.387*	*0.054*	*7.177*	*0.000*
PA	*0.240*	*0.043*	*5.517*	*0.000*
Diet	*0.425*	*0.060*	*7.043*	*0.000*

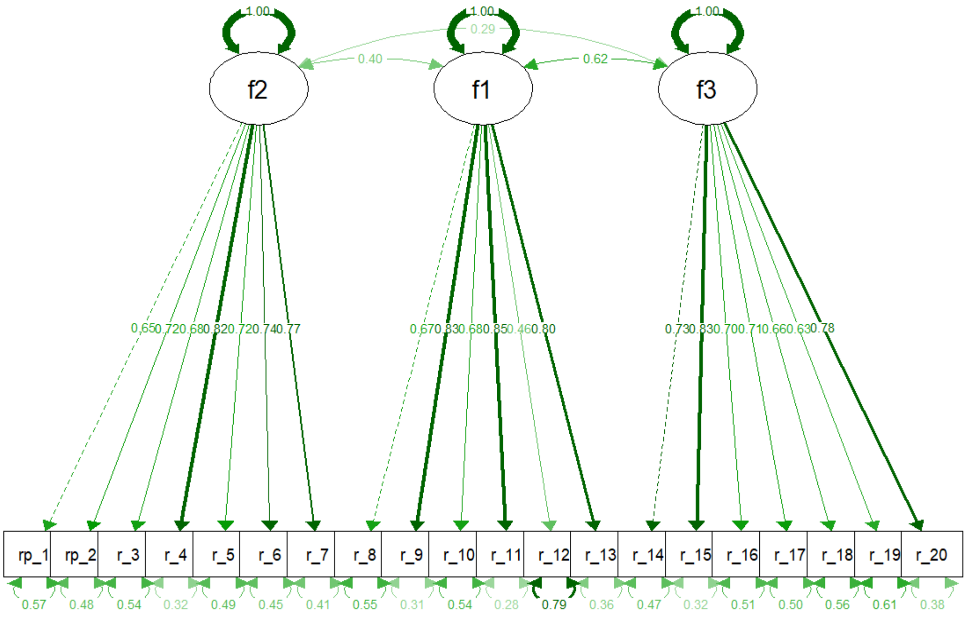

Figure S4. Confirmatory factor analysis: plot of the standardized estimates

Item properties - parametric Item Response Theory

Rasch and 2 – PL model: knowledge scale

Table S6 below presents a summary of the simple Rasch model (assuming equal discrimination of the items) and figure S5 displays the ICCs. The result show that question 9 is the easiest (% Correct = 92.0%; δ=-3.32; SE=0.18) and question 8 is the most difficult item (Correct=27.8%; δ=1.38; SE=0.12). On average, the respondents were located higher on the logit scale (M= -0.09, SD=1.67) compared to questions (M= -1.21; SD=1.23), suggesting that the knowledge items are relatively easy for respondents who participated in this survey. Average values of model-data fit statistics indicate that the model fit was adequate, with average Infit and Outfit mean square statistics (MSQ) around 1.00. The infit and outfit MSQs for all individual items were within the acceptable range, except for item 3, in which the outfit value was 0.53 (below 0.6), showing the item might be redundant from other items. Similarly, the 2-PL model showed the infit and outfit MNSQ for all the items were within the accepted range, except for the item 3, in which the infit value is 0.34 and the outfit value is 0.50 (below 0.6), showing the item might be redundant from other items.

Table S6. summary of the Rasch model parameters and item fit statistics

Item	% Correct	Delta (SE)	Outfit *MSQ*	Outfit_P	Infit *MSQ*	Infit_P
1	67.2	-0.67 (0.09)	0.88	0.29	1.05	0.64
2	68.4	-0.71 (0.09)	0.53	0.00	0.75	0.00
3	78.3	-1.19 (0.10)	1.29	0.00	1.17	0.01
4	62.1	-0.46 (0.08)	1.26	0.00	1.17	0.01
5	69.3	-0.76 (0.09)	0.87	0.01	0.98	0.74
6	74.1	-0.98 (0.10)	0.86	0.01	0.87	0.01
7	76.0	-1.06 (0.10)	0.92	0.12	1.02	0.66
8	27.8	-0.90 (0.15)	1.18	0.00	1.07	0.17
9	92.0	-2.17 (0.08)	0.83	0.00	0.95	0.42

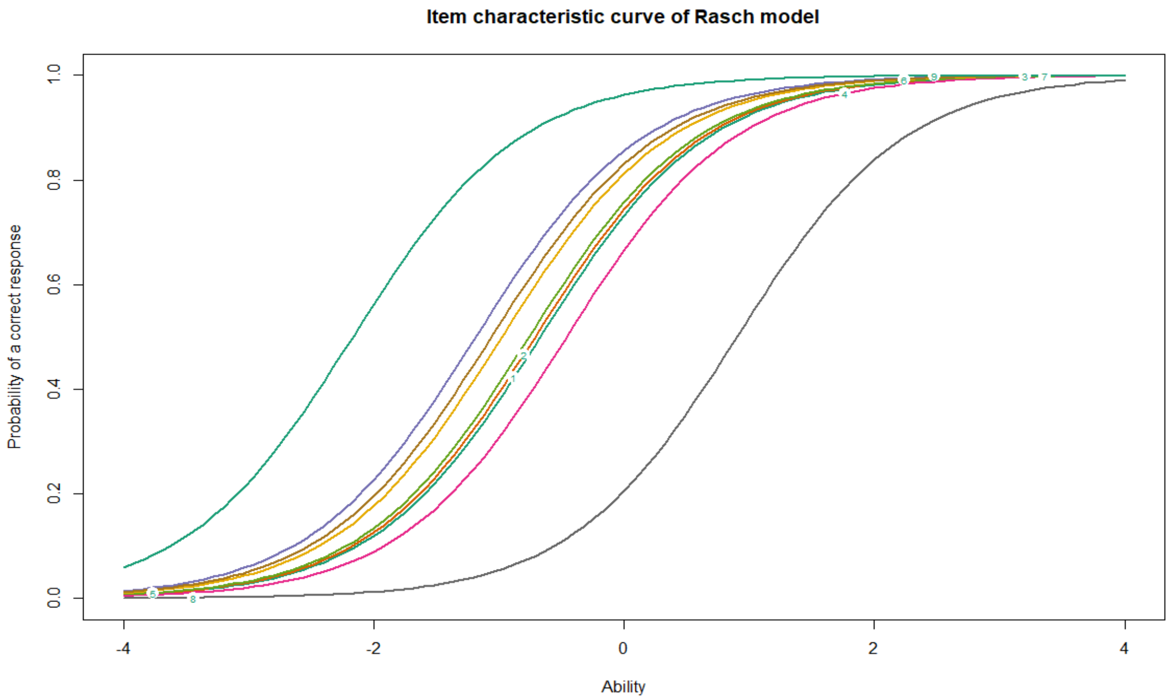

Figure S5. Joint ICCs of the Rasch model: ABCD-knowledge scale.

Comparing Rasch and 2-PL models

Since the two models are nested, we compared using a Likelihood-Ratio (LR) test. The test is significant (p <0 .001) indicating that both models do not fit equally well and that the more complex 2PL model is preferred. Further evaluation of differences between the two models, the difficulty parameters are correlated across models (r=0.94).

Likelihood Ratio Table

	AIC	BIC	Loglik	LRT	Df	p-value
Rasch	4681.41	4724.00	-2330.70			
2-PL	4563.46	4640.14	-2263.73	133.94	8	<0.001

Rating Scale Model – Risk scale

Table S7. Risk scale: Summary of item parameters and item fit of the RSM

Item	Mean	Severity (SE)	Outfit	Infit	τi.Cat1	τi.Cat2	τi.Cat3
**Risk perception**							
1	1.20	1.12 (0.09)	1.04	1.06	-2.65	1.14	4.88
2	1.13	1.42 (0.09)	0.86	1.88	-2.36	1.44	5.18
3	1.22	1.06 (0.09)	1.14	1.16	-2.72	1.07	4.82
4	0.97	2.05 (0.09)	0.79	0.79	-1.72	2.07	5.81
5	0.95	2.15 (0.09)	0.89	0.90	-1.63	2.16	5.91
6	1.13	1.42 (0.09)	1.11	1.09	-2.36	1.43	5.18
7	1.04	1.78 (0.09)	0.91	0.92	-2.00	1.79	5.54
**Perceived benefit and intention to change of PA**							
8	2.11	-2.03 (0.09)	1.28	1.13	-5.10	-2.31	1.31
9	2.08	-1.92 (0.09)	0.90	0.92	-4.98	-2.20	1.43
10	1.87	-1.13 (0.09)	0.62	0.66	-4.20	-1.41	2.21
11	1.89	-1.20 (0.09)	0.59	0.63	-4.27	-1.49	2.14
12	1.69	-0.49 (0.08)	1.24	1.24	-3.56	-0.77	2.85
13	1.91	-1.26 (0.09)	0.99	1.01	-4.32	-1.54	2.09
**Perceived benefit and intention to change of healthy dietary habit**							
14	1.92	-1.55 (0.09)	1.16	1.12	-5.21	-1.65	2.21
15	1.80	-1.06 (0.09)	0.82	0.83	-4.72	-1.17	2.69
16	1.68	-0.58 (0.09)	0.52	0.54	-4.24	-0.69	3.17
17	1.72	-0.74 (0.09)	0.60	0.62	-4.39	-0.84	3.02
18	1.60	-0.28 (0.09)	0.87	0.88	-3.93	-0.38	3.48
19	1.65	-0.46 (0.09)	1.36	1.36	-4.11	-0.56	3.30
20	1.74	-0.82 (0.09)	1.08	1.10	-4.48	-0.92	2.94

τi = Threshold of respective categories

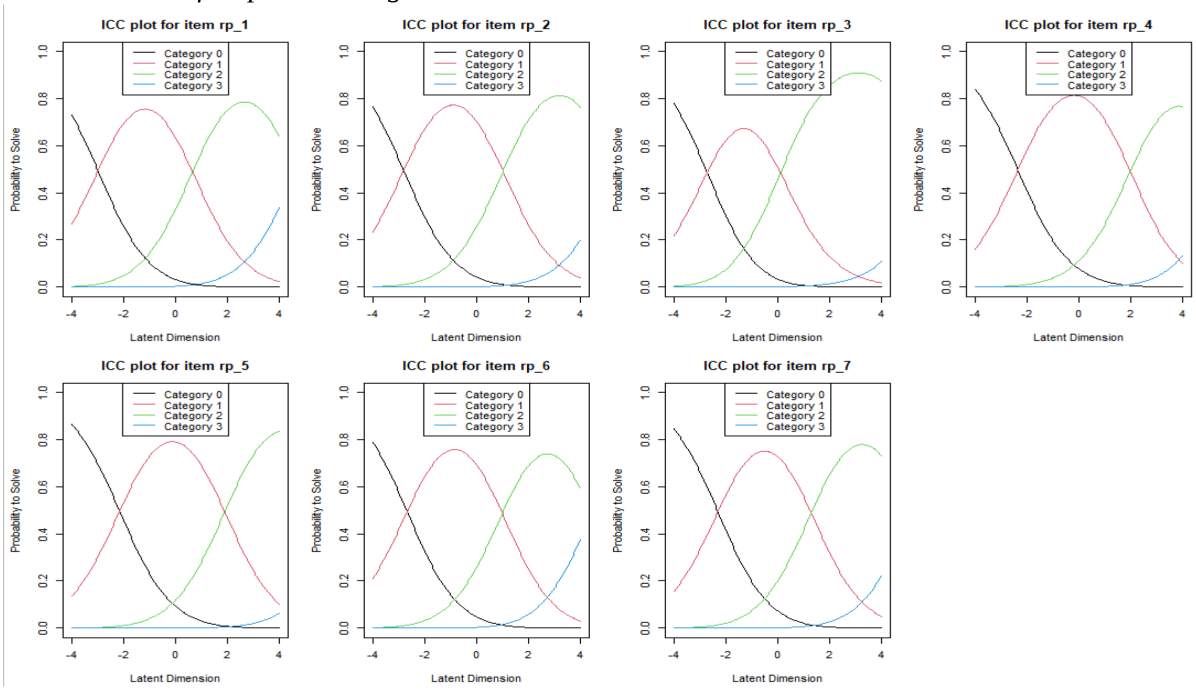

a.

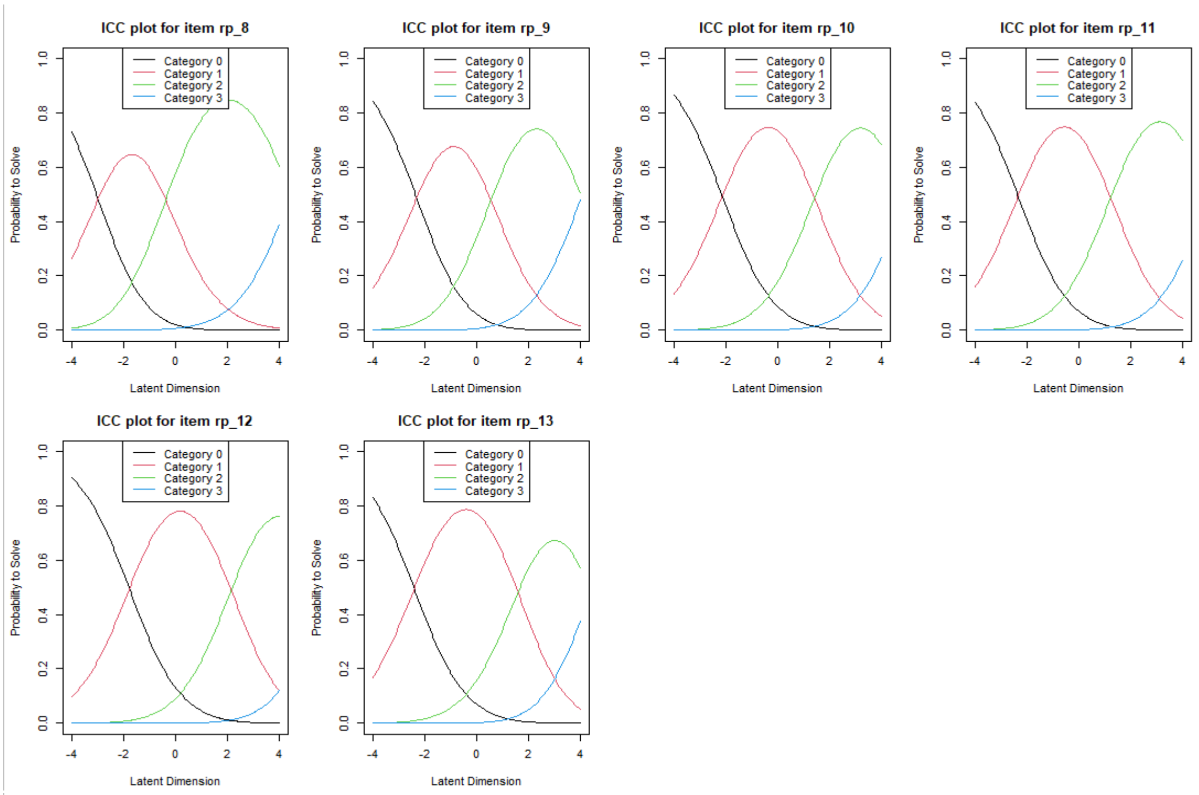

b.

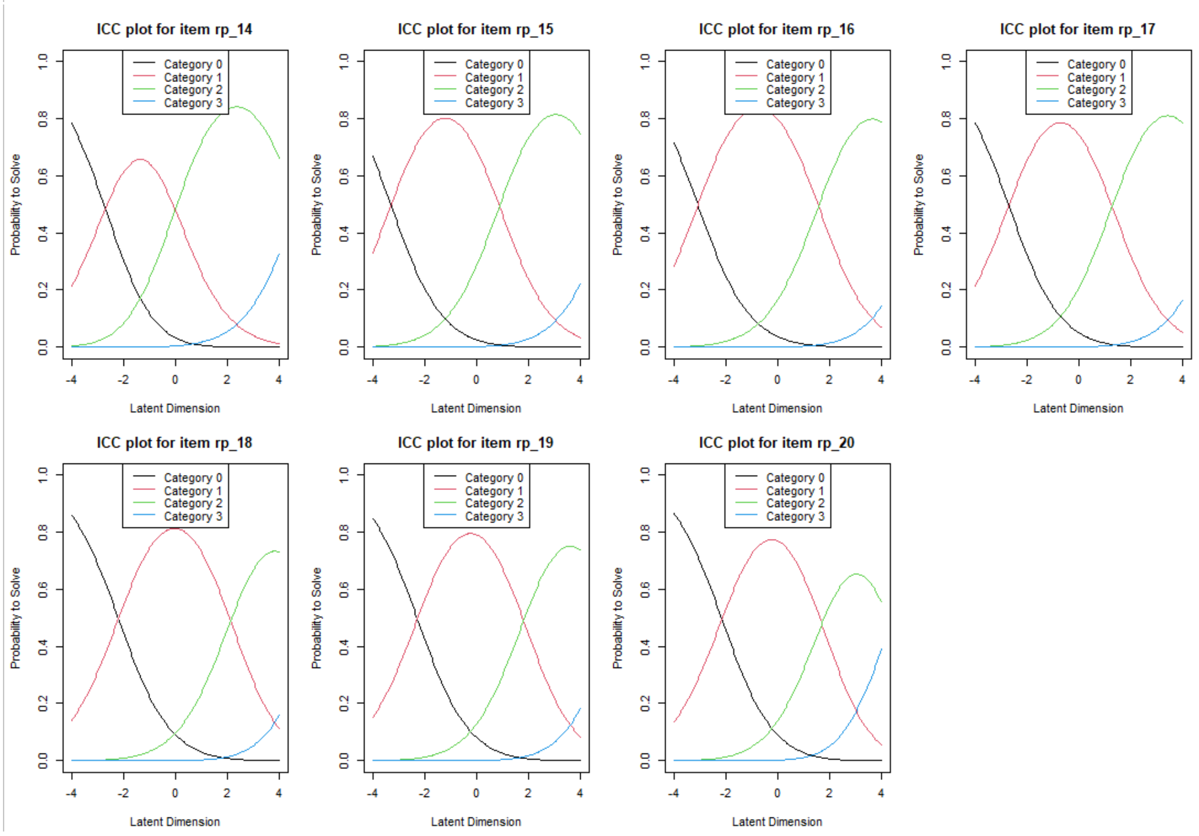

c.

Figure S6. RSM: Individual item characteristic curves of each domains of ABCD-risk scale (a) risk perception, b) perceived benefit and intention to change of PA and c) perceived benefit and intention to change of health diet)

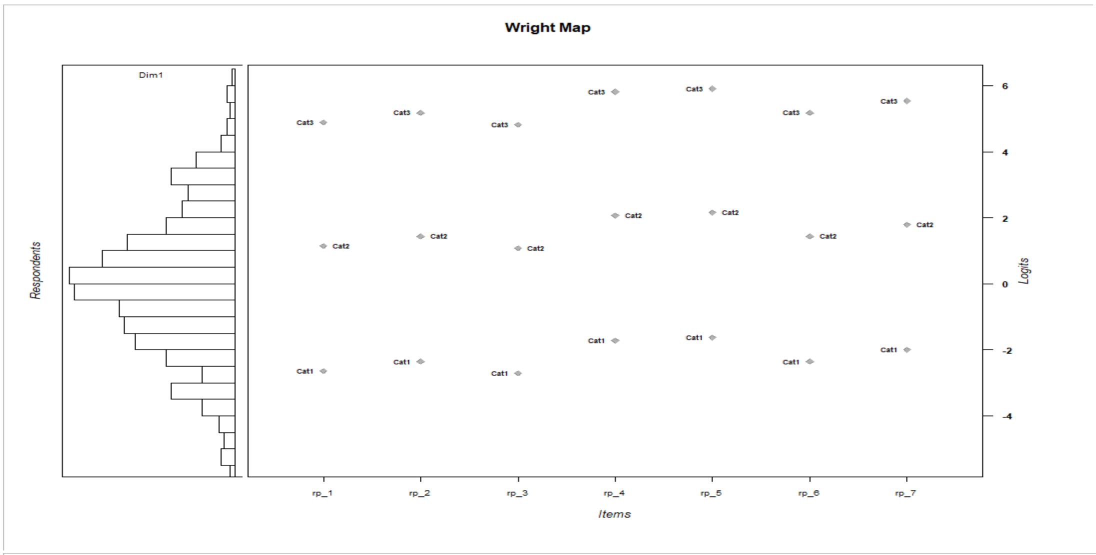

a.

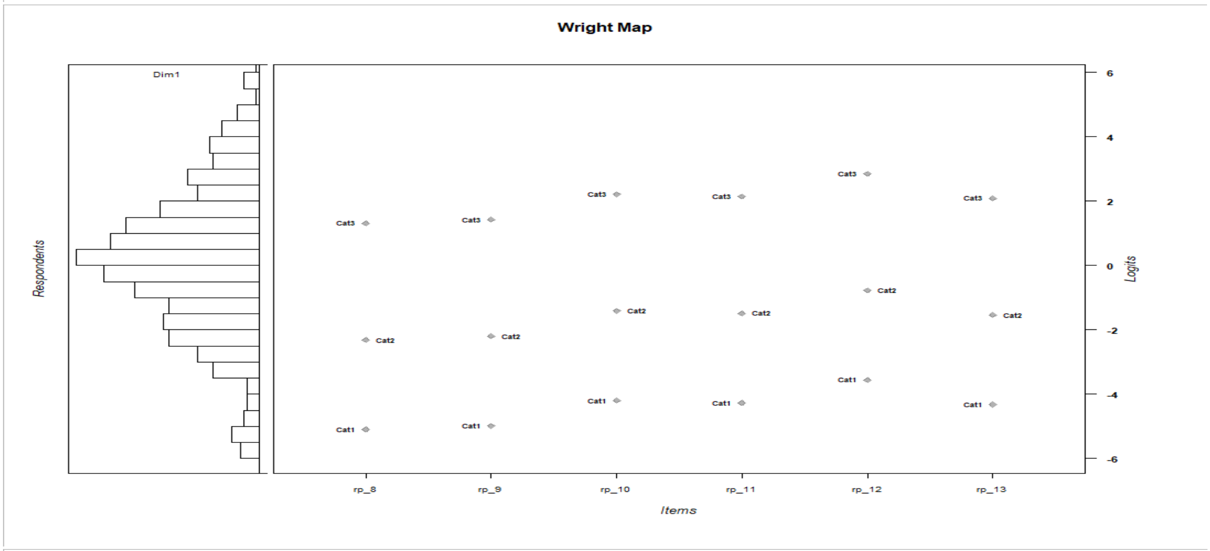

b.

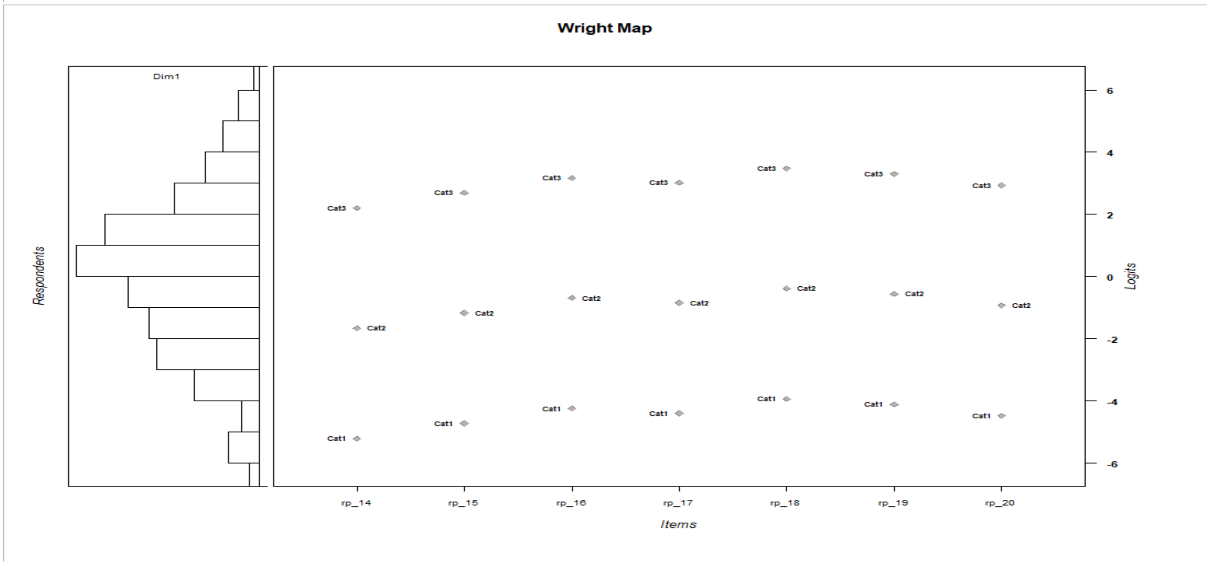

c.

Figure S7. RSM: Wright Map of each domain of ABCD-risk scale: (a) risk perception, b) perceived benefit and intention to change of PA and c) perceived benefit and intention to change of health diet)

Classical Test Theory analysis

Table S8. Knowledge scale – Reliability statistics if item dropped

Item	raw_alpha	std.alpha	G6(smc)	average_r	S/N	alpha se	var.r	med.r
question_1	0.730	0.730	0.749	0.252	2.698	0.017	0.020	0.228
question_2	0.707	0.708	0.751	0.232	2.420	0.019	0.022	0.200
question_3	0.695	0.695	0.726	0.221	2.276	0.020	0.017	0.201
question_4	0.737	0.734	0.774	0.257	2.765	0.017	0.023	0.229
question_5	0.723	0.724	0.744	0.247	2.628	0.018	0.020	0.228
question_6	0.751	0.749	0.799	0.271	2.981	0.016	0.030	0.244
question_7	0.767	0.753	0.823	0.272	2.986	0.016	0.027	0.228
question_8	0.745	0.744	0.799	0.257	2.765	0.017	0.031	0.212
question_9	0.750	0.752	0.800	0.275	3.027	0.017	0.029	0.249

Reliability statistics for the shorter form

Table S9. Summary of the reliability statistics for the shorter form of sub-scales

Constructs	Alpha	Gl6	Beta	Omega (95%CI)	Correlation with original
Knowledge	0.76	0.79	0.54	0.71 (0.65, 0.78)	0.951
Risk perception	0.91	0.92	0.83	0.91 (0.90, 0.93)	0.928
Perceived benefit and intention to change of physical activity	0.85	0.90	0.74	0.85 (0.84, 0.87)	0.962
Perceived benefit and intention to change of healthy eating	0.82	0.85	0.71	0.81 (0.80, 0.83)	0.947

Alpha: Cronbach's alpha; Gl6: Guttman's lambda6;

Dutch (Flemish) version of the ABCD questionnaire

Items in bold are included in the short version.

Kennis items<br>(Antwoordopties: 1) Juist, 2) Fout, and 3) Weet het niet)
**1. Stress is een van de hoofdoorzaken van hartaanvallen en beroertes.**<br>**2. Wandelen en tuinieren zijn activiteiten waarmee je het risico op een hartaanval of hartinfarct kan verminderen.**<br>**3. Als je 2,5 uur per week matig fysiek actief bent, vermindert jouw kans op een hartaanval of beroerte.**<br>**4. Mensen met diabetes hebben een verhoogd risico op een hartaanval of beroerte.**<br>**5. Door je stressniveau te beheersen, kan je je bloeddruk onder controle houden.**<br>6. Het drinken van grote hoeveelheden alcohol, kan jouw cholesterol en andere bloedvetten doen stijgen.<br>7. Er bestaat 'goede' cholesterol en er bestaat 'slechte' cholesterol.<br>**8. Het voorkomen van hartaandoeningen in de familie is geen risicofactor voor hoge bloeddruk.**<br>**9. Mensen die roken hebben een verhoogd risico op hart- en vaatziekten.**
**Risico perceptie items**<br>(Antwoordopties: 1) Helemaal niet eens, 2) Niet eens, 3) Eens, en 4) Helemaal eens
**1. Ik heb het gevoel dat ik ooit in mijn leven aan een hartaanval of beroerte zal lijden.**<br>**2. Het is waarschijnlijk dat ik in de toekomst een hartaanval of beroerte zal krijgen**<br>**3. Het is mogelijk dat ik binnen nu en tien jaar een hartaanval of beroerte zal krijgen.**<br>4. De kans dat ik binnen nu en tien jaar een hartaanval of beroerte krijg, is groot.<br>5. Ik zal waarschijnlijk een hartaanval of beroerte krijgen door mijn vroegere en/of huidige levensstijl.<br>**6. Ik maak me geen zorgen dat ik een hartaanval of beroerte zou kunnen krijgen.**<br>**7. Ik maak me zorgen omdat er een kans bestaat dat ik in de nabije toekomst een hartaanval of beroerte zal krijgen.**<br>**8. Wanneer ik minstens 5 maal 30 minuten per week aan lichaamsbeweging doe, dan doe ik iets goeds voor de gezondheid van mijn hart.**<br>**9. Het is belangrijk voor mij om regelmatig aan lichaamsbeweging te doen.**<br>**10. Ik overweeg om minstens 5 maal 30 minuten per week aan lichaamsbeweging te doen.**<br>**11. Ik ben van plan, of wil, minstens 5 maal 30 minuten per week aan lichaamsbeweging doen.**<br>**12. Ik ben er zeker van dat ik een gezond gewicht kan verkrijgen of behouden door minstens 5 maal 30 minuten per week aan lichaamsbeweging te doen in de komende 2 maanden.**<br>13. Ik overweeg niet om minstens 5 maal 30 minuten per week aan lichaamsbeweging te doen.<br>**14. Wanneer ik per dag ten minste 5 porties fruit en groenten eet, doe ik iets goeds voor de gezondheid van mijn hart.**<br>**15. Het is belangrijk voor mij om dagelijks minstens 5 porties groenten en fruit te eten.**<br>**16. Ik overweeg om per dag ten minste 5 porties fruit en groenten te eten.**<br>**17. Ik ben van plan, of wil, minstens 5 porties groenten en fruit eten per dag.**<br>**18. Ik ben er zeker van dat ik de komende 2 maanden ten minste 5 porties fruit en groenten per dag kan eten.**<br>19. Ik ben er zeker van dat ik een gezond gewicht kan verkrijgen of behouden door per dag minstens 5 porties groenten en fruit te eten in de komende 2 maanden.<br>20. Ik overweeg niet om per dag ten minste 5 porties fruit en groenten te eten.
